# Association of Nap Duration after Lunch with Prevalence of Metabolic Syndrome in a Chinese Government Employee Population

**DOI:** 10.3390/ijerph17124268

**Published:** 2020-06-15

**Authors:** Jun He, Feiyun Ouyang, Dan Qiu, Yanying Duan, Dan Luo, Shuiyuan Xiao

**Affiliations:** 1 Department of Social Medicine and Health Management, Xiangya School of Public Health, Central South University, Changsha 410078, China; hejunjason@csu.edu.cn (J.H.); feiyun0716@foxmail.com (F.O.); qiudan@csu.edu.cn (D.Q.); luodan_csu_2011@126.com (D.L.); 2 Department of Occupational and Environmental Health, Xiangya School of Public Health, Central South University, Changsha 410078, China; duany@csu.edu.cn

**Keywords:** nap, metabolic syndrome, mood symptom, government employee, China

## Abstract

Metabolic syndrome is an important risk factor for cardiovascular disease, type 2 diabetes mellitus, and all-cause mortality. However, the relationship between napping and metabolic syndrome remains contradictory. The aim of this study was to evaluate the association between nap duration after lunch and prevalence of metabolic syndrome, and subgroup differences in the associations among a government employee population. A total of 5129 participants (mean age 39.4 years) were included in this study. Nap duration after lunch was self-reported, and metabolic syndrome was defined according to the Joint Interim Statement in 2009. Multivariate logistic regression models and adjusted restricted cubic spline functions were used to examine the association and dose-response relationship between nap duration after lunch and prevalence of metabolic syndrome. Of the participants, 17.3% had metabolic syndrome and 81.4% were habitual nappers. Adjusted odds ratio (OR) (95% confidence interval (CI)) of metabolic syndrome for longer nap duration (>90 min) per day was 1.77 (95% CI 1.09 to 2.89), compared with reference (~30 min). Results of stratification analyses indicated the association was found only among females (OR 3.02, 95% CI 1.38 to 6.57), participants without mood symptoms (OR 1.97, 95% CI 1.19 to 3.25), and those having longer night sleep duration (≥8 h) (OR 2.34, 95% CI 1.20 to 4.56). Longer nap duration was also associated with components of metabolic syndrome, including elevated waist circumference, elevated fasting plasma glucose, and elevated triglycerides. In conclusion, longer nap duration after lunch was associated with a higher prevalence of metabolic syndrome in females, people without mood symptoms, and people having longer night sleep duration.

## 1. Introduction

Metabolic syndrome is a constellation of metabolic abnormalities including central obesity, dyslipidemia, hyperglycemia, and hypertension [1,2,3]. It is an important risk factor for cardiovascular disease, type 2 diabetes mellitus, and all-cause mortality [4,5]. In the Americas, Europe, and India, at least one-fourth of adults suffer from this disorder [6]. In China, the prevalence of metabolic syndrome varies from 17.8% to 36.8% in females and 9.8% to 31.0% in males, depending on the criteria adopted [7,8,9,10,11]. Metabolic syndrome has become a serious public health problem, and enough attention should be given to its associated factors to prevent the syndrome.

Napping (siesta) is a ubiquitous behavior across the human lifespan. In China, napping around noon, which is also called *wujiao* in Chinese, is considered part of a healthy lifestyle and has been embedded into the culture [12]. A nap may offer benefits such as sleepiness reduction, memory consolidation, cognitive performance enhancement, boost in emotional stability, and endurance performance improvement [13,14,15]. However, in spite of the reported benefits, longer duration napping has also been associated with many negative outcomes. A study of older females in United States reported daily napping significantly increased the risk of death [16]. Moreover, longer day nap was associated with a risk of type 2 diabetes, hypertension, and cardiovascular disease, independent of covariates such as demographic characteristics, lifestyle, sleep quality, disease history, and family history [17,18,19]. In addition to outcomes related to metabolic syndrome, longer nap duration was also believed to increase risk of osteoporosis and renal hyperfiltration [20,21].

Association between nap duration and metabolic syndrome has been reported in several previous studies. Lin et al. conducted a cross-sectional study of 8547 subjects with a mean age of 56 years and reported that a nap of more than 60 min was associated with a higher prevalence of metabolic syndrome only in females [22]. The cross-sectional evidence from the Dongfeng-Tongji retiree cohort study suggested that longer daytime napping (≥90 min) was associated with metabolic syndrome in females [23,24]. Recently, a study of 1679 older adults by van der Pal et al. found a relationship different from the previous studies. They linked both daytime naps of ≤30 min and >30 min to metabolic syndrome, compared with those who did not nap [25]. In addition, a meta-analysis in 2016 showed that longer nap duration (≥60 min) was associated with a higher risk of metabolic syndrome and revealed a J-curve relation; however, the analysis included only two articles and pooled odds ratios (ORs) from different nap duration group (>60 and ≥90 min) into one [26]. The contradictory results can be attributed to differences in the populations, ages, and classifications of nap duration in these studies. The risk of prolonged napping may vary across occupations and age groups, and we cannot ascertain the risk of 61~90 min nap group if the >60 min group was not split. More detailed evidence from wider populations and age groups is needed to resolve these contradictions.

Government employees are physically inactive and therefore more susceptible to chronic diseases than the general population [27,28]; however, the relationship between napping and metabolic syndrome in this population is little known. Therefore, the present study conducted analyses aimed to find out the association of nap duration after lunch with prevalence of metabolic syndrome, and to assess their dose-response relationship and the subgroup differences in a Chinese government employee population.

## 2. Materials and Methods

### 2.1. Study Population

This paper presents a cross-sectional study in Changsha City, Hunan Province, which aimed to investigate chronic diseases in the government employee population. In China, government employees mainly include civil servants in government departments, employees of public institutions, and employees of state-owned enterprises. Government departments are organizations that exercise state authority and perform state administrative functions according to law; public institutions are social service organizations, such as universities and hospitals, set up by the government and engaged in education, science and technology, culture, health, etc.; and state-owned enterprises refer to commercial companies fully or largely controlled by the government.

From January 2018 to November 2018, a total of 6414 employees (21–60 years old) from 10 government organizations who agreed to answer the questionnaire online and performed health examinations were recruited via cluster sampling consecutively at the Health Management Center of the Third Xiangya Hospital. A digital self-reported questionnaire platform was established to collect information on participants’ nap and covariates. Recruited participants accessed the questionnaire with URLs sent by Short Messaging Service (SMS) and answered the questions via phone, tablet, or PC. Individuals with self-reported coronary heart disease or stroke (*n* = 19) and those who did not complete the survey or those with missing values on nap duration, metabolic syndrome components, or covariates (*n* = 1266) were excluded stepwise, leaving 5129 participants for the final analyses. Compared to characteristics of participants in the analyses, those excluded were more likely to be male, from government departments, with lower positions, higher rates of drinking, higher levels of waist circumference, higher levels of triglycerides, higher levels of high-density lipoprotein cholesterol (HDL-C), and higher levels of blood pressure, while fasting plasma glucose and other features were not statistically different between the two groups (Supplementary Table S1).

### 2.2. Ethics Approval and Informed Consent

The study was approved by the Ethics Committee of Xiangya School of Public Health, Central South University (No. XYGW-2016-10). Informed consent was obtained from all participants.

### 2.3. Assessment of Nap Duration

By asking the question, “If you have a habit of napping after lunch in the last six months, what is the average duration of your naps in minutes? (Fill in 0 if you don’t nap),” we collected the nap duration of participants and grouped them by every 30 min, creating the following groups: 0 min, ~30 min, ~60 min, ~90 min, and >90 min. We set the nap duration of ~30 min as a reference group.

### 2.4. Definition of Metabolic Syndrome

Metabolic syndrome was defined according to the Joint Interim Statement in 2009 [29,30]. Participants with three or more of the following abnormities can be diagnosed as metabolic syndrome:

(1) Elevated waist circumference: ≥85 cm in males or ≥80 cm in females;
(2) Elevated triglycerides (drug treatment for elevated triglycerides is an alternate indicator): ≥150 mg/dL (1.7 mmol/L);
(3) Reduced HDL-C (drug treatment for reduced HDL-C is an alternate indicator): <40 mg/dL (1.0 mmol/L) in males or <50 mg/dL (1.3 mmol/L) in females;
(4) Elevated blood pressure (antihypertensive drug treatment in a patient with a history of hypertension is an alternate indicator): systolic blood pressure ≥130 and/or diastolic blood pressure ≥85 mm Hg;
(5) Elevated fasting plasma glucose (drug treatment of elevated glucose is an alternate indicator): ≥100 mg/dL (5.6 mmol/L).

### 2.5. Measurement Methods

The information needed to define metabolic syndrome was obtained from the measurements taken during a health examination. Waist circumference was measured at the midpoint between the lower rib and upper margin of the pelvic bone by a trained nurse using a tape. Sitting blood pressure was measured by a skilled physician using a corrected mercury sphygmomanometer after participants had rested for 15 min. Systolic blood pressure and diastolic blood pressure were measured three times, with a 30 s interval, and the average of three readings was calculated for recording. Blood pressure will be re-measured if the difference between the three measurements was greater than 5 mmHg. Blood samples were collected at 07:30–10:00 after a fasting period of 12 h and were stored at −20 °C until tested. Triglycerides and fasting plasma glucose levels were measured by enzymatic colorimetric method, and HDL-C concentration was determined by lipoprotein electrophoresis, both using a Chemistry system Autoanalyzer (Hitachi 7600-110; Tokyo, Japan) in the Medicine Laboratory Department of the Third Xiangya Hospital, which has been accredited by the Chinese Society of Laboratory Medicine.

### 2.6. Covariates

Covariates in this study included participants’ demographics, lifestyle habits, mood symptoms, sleep-related conditions, family histories, and diets, which were derived from the digital self-reported questionnaire.

Demographic factors included age, gender, affiliation type, marital status, and position level. Affiliations of participants were categorized as government department, public institution, and state-owned enterprise. Marital status was divided into married/cohabitating, unmarried, and divorced/widowed. Position levels were classified into primary title/staff member/clerk, intermediate title/section level, and senior title/division level or above.

Lifestyle habits included smoking status, drinking status, and physical activity. Smoking status was divided into current smoking and non-current smoking (including former smoking and non-smoking). Those who smoked at least one cigarette a day for more than six months were considered as current smoking. Drinking was dichotomized into current drinking versus non-current drinking, which included ever drinking and never drinking. Participants who drank alcohol at least once a week for at least six months were defined as current drinking. Physical activity was categorized into participation and non-participation, with the former having to exercise more than once a week on average.

We used depression and anxiety conditions to represent mood symptoms. Depression and anxiety were evaluated by Patient Health Questionnaire-2 (PHQ-2) and General Anxiety Disorder-2 (GAD-2), respectively. Participants with a score of three or more for PHQ-2 and/or GAD-2, or with a self-reported depression or anxiety diagnosis were considered to have mood symptoms [31,32].

Sleep-related conditions were measured using items of the Pittsburgh sleep quality index (PSQI) [33]. Night sleep duration was calculated as the interval between usual bedtime and usual getting-up time. Use of sleeping medication was grouped into using and not using during the past month. Night sleep quality was classified into good, fair, and bad.

In addition, a family history of hypertension, cancer, or diabetes mellitus was considered to be present if either grandparents, parents or sibling had the disease. Diet frequency of coarse cereals, meat, poultry, aquatic products, egg products, vegetables, dairy products, fruits, and dessert was divided into the following five groups: daily, 4–6 days a week, 1–3 days a week, less than once a week, and rarely or never.

### 2.7. Statistical Analyses

The mean and standard deviation (SD) or proportion (%) of covariate characteristics were presented among participants with or without metabolic syndrome. Student *t*-tests were used for numerical variables to evaluate the differences of characteristics between groups, and chi-square tests were used for categorical variables. Logistic regression models were used to estimate ORs and corresponding 95% confidence intervals (CIs) of metabolic syndrome for each nap duration. In the main analysis, the full model was adjusted for demographic factors (age, gender, affiliation type, marital status, and position level), lifestyle habits (smoking status, drinking status, and physical activity), mood symptoms, and sleep-related covariates (night sleep duration hours, bedtime at night, use of sleeping medicine, and sleep quality).

To examine associations in different participants, analyses were explored across subgroups stratified by gender, mood symptoms, and night sleep duration (<7 h, 7~ h, and 8~ h). Furthermore, multivariable restricted cubic spline (RCS) functions with logistic regression models were performed to explore the curvilinear dose-response relationship of nap duration and prevalence of metabolic syndrome visually, with five knots located at nap duration quantiles of 5%, 27.5%, 50%, 72.5%, and 95% [34]. Logistic regression models were also used to test the relationships between nap duration and components of metabolic syndrome. Subgroup, RCS and components regression analyses were all fitted with the same covariates as in the main analysis.

In sensitivity analyses, four procedures were conducted to examine the robustness of the results. First, in addition to covariates in the main analysis, family history and diet frequency factors were further controlled, including family history of hypertension, cancer, and diabetes mellitus, and weekly frequency of eating coarse cereals, meat, poultry, aquatic products, egg products, vegetables, dairy products, fruits, and dessert. Second, because the method of dealing with missing data in analyses was listwise deletion and information of deleted data was not utilized, multivariate imputation by chained equations was further used, and the association between nap duration after lunch and prevalence of metabolic syndrome was reevaluated by pooling the results of five imputed data sets [35]. Third, we performed a multilevel logistic regression model, in which the individuals were level 1 and the government organizations were level 2, to account for different intercepts of 10 affiliations in regression. Fourth, we also adjusted potential confounders through propensity score stratifying analysis, in which propensity scores were calculated by logistic regression with dependent variable dichotomized by whether nap duration was greater than 90 min and were divided into six strata.

All analyses and plots were performed with packages “base” (version 4.0.0), “stats” (4.0.0), “tableone” (0.11.1), “rms” (5.1–4), “mice” (3.8.0), “lme4” (1.1–23), “MatchIt” (3.0.2), “forestplot” (1.9), “ggplot2” (3.3.0), and “ggpubr” (0.3.0) in R. *P* values < 0.05 (two-sided tests) were considered statistically significant.

## 3. Results

### 3.1. Characteristics of Participants

Of 5129 participants, the mean (SD) age was 39.4 (9.3) years, 59.0% were female, 17.3% had metabolic syndrome, and 81.4% were habitual nappers with a mean (SD) duration of 38.1 (25.6) minutes. Table 1 presents characteristics of study population according to whether they had metabolic syndrome. Subjects with metabolic syndrome tended to be older, male, married or cohabitating, and were more likely to have higher position and longer nap duration after lunch than those without metabolic syndrome. Besides, subjects with nap duration of >90 min had the highest prevalence of metabolic syndrome among all nap duration groups (Supplementary Table S2).

### 3.2. Association between Nap Duration after Lunch and Prevalence of Metabolic Syndrome

**Figure 1:**
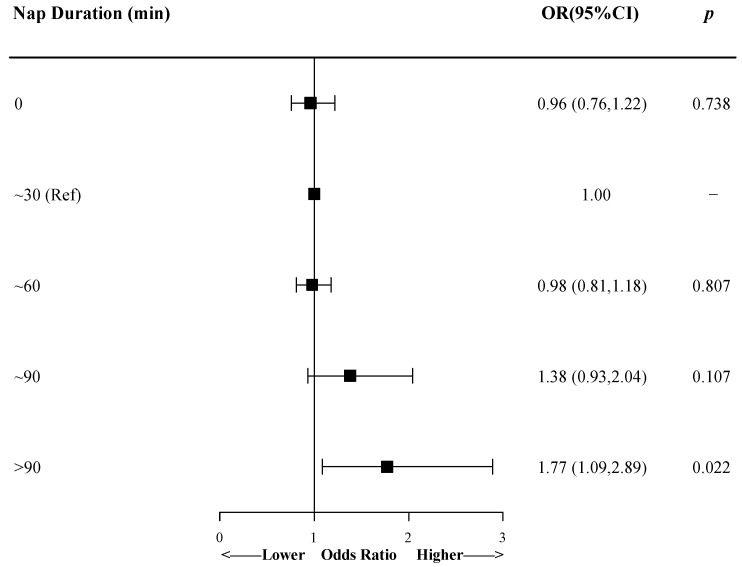
The association between nap duration and prevalence of metabolic syndrome by logistic regression model. Model was adjusted for demographic factors, lifestyle habits, mood symptoms, and sleep-related characteristics. Small squares represent the point estimates of odds ratios, and horizontal lines represent 95% CIs. OR, odds ratio; CI, confidence interval; Ref, reference.

Figure 1 shows the association between nap duration after lunch and prevalence of metabolic syndrome when the duration was grouped by every 30 min. After adjusting for demographic factors, lifestyle habits, mood symptoms, and sleep-related characteristics, the OR of metabolic syndrome for participants with nap duration of >90 min was 1.77 (95% CI 1.09 to 2.89), compared with those with nap duration of ~30 min. The results were similar when fewer covariates were included (Supplementary Figure S1).

**Figure 2:**
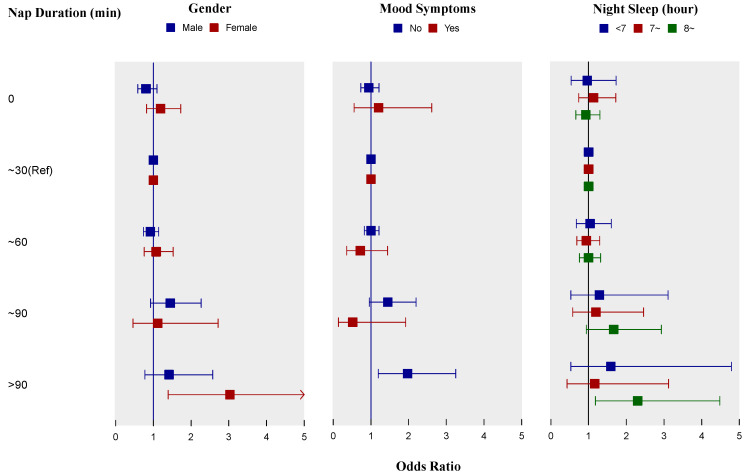
Subgroup analyses of associations between nap duration and prevalence of metabolic syndrome by logistic regression models according to gender, mood symptoms, and night sleep hours. All three models were adjusted for age, affiliation, marital status, position level, smoking status, drinking status, physical activity, bedtime at night, use of sleeping medicine, and sleep quality, and were adjusted for gender, mood symptoms, and night sleep duration as appropriate. In subgroup analysis of participants with mood symptoms, the odds ratio for group of >90 min was absent because no participant in this group had metabolic syndrome. Small squares represent the point estimates of odds ratios and horizontal lines represent 95% confidence intervals. Ref, reference.

The subgroup analyses according to gender found a similar relationship in females, and the OR for longer nap duration (>90 min) obviously increased to 3.02 (95% CI 1.38 to 6.57), but the association was not observed in males (Figure 2). Moreover, females who did not nap had a higher OR (1.19, 95% CI 0.82 to 1.72) than males, though the difference was not significant. When the analysis was restricted to subjects without mood symptoms, the OR for >90 min group mildly increased to 1.97 (95% CI 1.19 to 3.25). However, it was not significant among participants with symptoms of anxiety and/or depression, and indeed the OR seemed to decrease as the nap duration increased. The association was significant only in people with ≥8 hours sleep at night (OR 2.34, 95% CI 1.20 to 4.56) when subgroup analyses were stratified by night sleep duration. There was no interaction between nap duration and gender, mood symptoms, or nighttime sleep duration (*p* > 0.05 for interaction).

**Figure 3:**
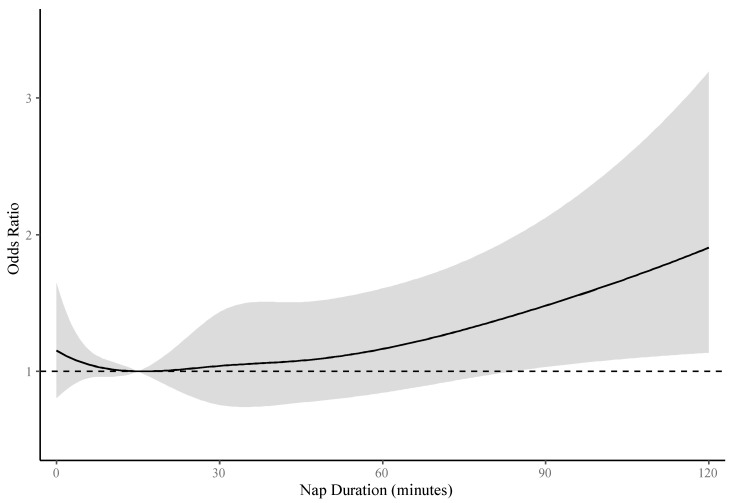
The dose-response relationship between nap duration and prevalence of metabolic syndrome. The curve was estimated by restricted cubic spline function with five knots and logistic regression model adjusted for age, gender, affiliation, marital status, position level, smoking status, drinking status, physical activity, mood symptoms, night sleep duration hours, bedtime at night, use of sleeping medicine, and sleep quality. The reference was set to 15 min. The shadow represents 95% confidence intervals of odds ratios. The dotted line represents level of odds ratio equal to 1.

Figure 3 displays multivariate adjusted restricted cubic spline curve of all participants—the OR initially decreased and reached its lowest at a nap of about 15 min, and then it increased slightly until about 45 min, followed by a relatively rapid increase at longer nap duration. Females had a similar trough around 15 min, while ORs for all nap durations were not significant among males (Supplementary Figure S2).

### 3.3. Associations between Nap Duration after Lunch and Metabolic Syndrome Components

Table 2 lists the multivariable logistic regression results of the associations between nap duration after lunch and metabolic syndrome components. For all participants, compared with ~30 min group, ORs of elevated waist circumference and elevated fasting plasma glucose for longer nap duration (>90 min) were 1.55 (95% CI 1.02 to 2.35) and 1.59 (95% CI 1.02 to 2.47), respectively. Of elevated triglycerides, ~90 min group also had an OR (1.48, 95% CI 1.04 to 2.11) with statistical significance, while associations of other components were not significant. For females, longer nap duration (>90 min) was associated with elevated waist circumference (OR 2.12, 95% CI 1.22, 3.70) and elevated fasting plasma glucose (OR 2.53, 95% CI 1.41, 4.53). For males, only ~90 min group had a statistically significant OR (1.87, 95% CI 1.21, 2.91) of elevated triglycerides.

### 3.4. Sensitivity Analyses

The results of the sensitivity analyses showed that the association of longer nap duration (>90 min) with metabolic syndrome was consistent before (OR 1.77, 95% CI 1.09 to 2.89) and after (OR 1.79, 95% CI 1.08 to 2.96) additional adjusting for family history and diet frequency. It also did not change substantially in multiple imputation (OR 1.69, 95% CI 1.06 to 2.69) and multilevel model (OR 1.73, 95% CI 1.06 to 2.82). After adjusting confounders by propensity score, the OR of metabolic syndrome for >90 min nap group was 1.65 (95% CI 1.08–2.52), compared with 0~90 min group (Supplementary Table S3).

## 4. Discussion

The present study suggested that longer nap duration after lunch (>90 min) was associated with a higher prevalence of metabolic syndrome (OR 1.77, 95% CI 1.09 to 2.89). Subgroup analyses suggested the association was found only in females (OR 3.02, 95% CI 1.38 to 6.57), participants without mood symptoms (OR 1.97, 95% CI 1.19 to 3.25), and those having longer night sleep duration (OR 2.34, 95% CI 1.20 to 4.56). The nap duration with a lower prevalence of metabolic syndrome appeared to be less than 45 min. Our findings also indicated that longer nap duration was associated with the components of metabolic syndrome, including elevated waist circumference, elevated fasting plasma glucose, and elevated triglycerides.

Napping is traditionally believed to be a good habit and beneficial to health; however, recent studies have shown that longer naps can be harmful. A result pooled by Yamada et al. from 11 cohort studies showed that napping for at least 60 min a day was associated with higher incidence of cardiovascular disease and elevated all-cause mortality compared with not napping [36]. Daytime napping also tended to be a risk factor of diabetes mellitus and hypertension [17,18,37]. Several studies have examined the relationship between daytime nap and risk of metabolic syndrome. The main finding of the present study was consistent with the cross-sectional result of the Dongfeng-Tongji retiree cohort study that napping more than 90 min was associated with a higher prevalence of metabolic syndrome only in females [23]. However, our results have not found a relationship between <90 min nap and metabolic syndrome yet, in contrast to studies conducted by Lin et al. and van der Pal et al. [22,25]. The five-year follow-up study of the Dongfeng-Tongji cohort did not performed gender-specific analyses, and there was no statistically significant association observed between nap and the incidence of metabolic syndrome components [24]. Overall, this study suggested that only longer nap duration after lunch was associated with metabolic syndrome prevalence rather than any duration.

The biological mechanisms linking longer nap duration to metabolic syndrome remain unclear. However, there are several possible speculations. Mistimed sleep during the daytime might induce circadian misalignment, and sequentially result in metabolic and endocrine abnormalities, leading to obesity, hyperglycemia, high triglycerides, and insulin resistance, the major role in metabolic syndrome development [38,39,40]. Furthermore, longer sleep duration may also be associated with an increase in levels of systemic inflammatory markers (e.g., C-reactive protein, interleukin-6) [41]. These markers can contribute to blood pressure, waist circumference, and insulin sensitivity and then form metabolic syndrome [42,43,44,45]. In addition, sleep apnea may prolong a nap after lunch, and 50–60% of people with metabolic syndrome also suffer from obstructive sleep apnea syndrome. Sleep apnea can lead to insulin resistance, endothelial dysfunction, and increased arterial rigidity with mechanisms such as sympathetic hyperactivity, inflammation, oxidative stress, and disruption of cortisol secretion [46].

In subgroup analyses of this study, the association was found only in females, participants without mood symptoms, and longer nighttime sleep group (≥8 h), but not in other groups. Males and females have different types and levels of sex hormones, and their bodies respond differently to them. Those may result in gender differences in glucose homeostasis, prediabetic syndromes and progression of diabetes [47]. The discrepancies in two mood symptoms subgroups can share the same reason as those in nighttime sleep duration subgroups. Anxiety and depression are associated with sleep disturbance [48,49], so the night sleep of those with mood symptoms may be shorter and worse than others. This could attenuate the effect of napping in causing metabolic syndrome, though the duration and quality of night sleep were adjusted to some extent in this study.

The present study added to the evidence of association and dose-response relationship between nap duration and prevalence of metabolic syndrome in government employee population. However, there still exist several potential limitations. First, this was a cross-sectional study that could not ascertain the sequence of excessive napping after lunch and metabolic syndrome emerging. Napping could be a sign or manifestation of poor health, which unfortunately was not evaluated in this study. Thus, causality needs to be confirmed in cohort studies. Second, self-reported nap duration after lunch in this study was not as accurate as objective measurement. Moreover, the employed population may not tell the truth about long naps, especially after lunch, which would underestimate the association. Third, although we have adjusted for a large range of covariates in analyses, uninvolved residual confounders such as preclinical disease, sleep apnea, and other sleep disorder could still introduce biases and obscure results. Fourth, we could not ascertain the OR for those with mood symptoms and napping more than 90 min in subgroup analysis, although there appeared to be a negative correlation between napping duration and prevalence of metabolic syndrome. This need to be further determined by larger sample studies. Finally, the study was conducted among government employee adults in China, who were characterized by prolonged sedentary time, lack of physical activity, relatively high prevalence of chronic diseases, and high level of fatigue. This population may be heterogeneous with general employees and other populations. There may also be heterogeneity among the 10 government organizations in this study. Therefore, caution is needed when extrapolating the findings to other populations. The main strengths of this study included the relatively large sample of middle-aged individuals, adjusting for a large number of covariates, and the online questionnaire platform. We were the first to perform subgroup analyses by emotional symptoms and to use RCS analyses to show the dose-response relationship between nap duration after lunch and metabolic syndrome. Moreover, four sensitivity analyses were conducted to verify the value and reliability of the results.

## 5. Conclusions

In summary, this study demonstrated that napping for more than 90 min per day was associated with an increased prevalence of metabolic syndrome in a Chinese government employee population. In subgroup analyses, the association was found only in females, participants without mood symptoms, and those having longer night sleep duration. People napping less than 45 min seemed to have a lower prevalence of metabolic syndrome. The longitudinal relationship between nap duration and risk of metabolic syndrome needs to be further confirmed in this population.

## Figures and Tables

**Table 1:** Characteristics of participants according to metabolic syndrome.

Characteristics	Non-Metabolic Syndrome	Metabolic Syndrome	*p*
N	4244	885	
Age (years), mean (SD)	38.35 (8.96)	44.59 (9.26)	<0.001
Gender, female (%)	2796 (65.9)	229 (25.9)	<0.001
Affiliations (%)			<0.001
Government department	265 (6.2)	105 (11.9)	
Public institution	2962 (69.8)	470 (53.1)	
State-owned enterprise	1017 (24.0)	310 (35.0)	
Marital status (%)			<0.001
Married/cohabitating	3534 (83.3)	815 (92.1)	
Unmarried	592 (13.9)	42 (4.7)	
Divorced/widowed	118 (2.8)	28 (3.2)	
Position levels (%)			<0.001
Primary title/staff member/clerk	1605 (37.8)	252 (28.5)	
Intermediate title/section level	1753 (41.3)	350 (39.5)	
Senior title/division level or above	886 (20.9)	283 (32.0)	
Current smoking (%)	376 (8.9)	236 (26.7)	<0.001
Current drinking (%)	202 (4.8)	152 (17.2)	<0.001
Participating physical activity (%)	2422 (57.1)	563 (63.6)	<0.001
Having mood symptoms (%)	459 (10.8)	67 (7.6)	0.005
Night sleep duration (h), mean (SD)	7.56 (1.06)	7.50 (0.98)	0.131
Using sleeping medication (%)	158 (3.7)	28 (3.2)	0.477
Night sleep quality (%)			<0.001
Good	1817 (42.8)	404 (45.6)	
Fair	1915 (45.1)	420 (47.5)	
Bad	512 (12.1)	61 (6.9)	
Nap duration (min), mean (SD)	30.46 (27.15)	33.67 (28.59)	0.002
WC (cm), mean (SD)	76.32 (8.42)	89.85 (7.18)	<0.001
TG (mmol/L), mean (SD)	1.12 (0.73)	2.85 (2.49)	<0.001
HDL-C (mmol/L), mean (SD)	1.48 (0.30)	1.13 (0.22)	<0.001
SBP (mmHg), mean (SD)	114.26 (12.16)	129.68 (13.70)	<0.001
DBP (mmHg), mean (SD)	69.12 (9.28)	81.10 (10.64)	<0.001
FPG (mmol/L), mean (SD)	5.20 (0.63)	6.20 (1.77)	<0.001

Data were indicated as mean (standard deviation) or number (percentage). *p* was calculated using *t*-test for continuous variables and chi-square test for categorical variables. SD, standard deviation; WC, waist circumference; TG, triglycerides; HDL-C, high-density lipoprotein cholesterol; SBP, systolic blood pressure; DBP, diastolic blood pressure; FPG, fasting plasma glucose.

**Table 2:** Adjusted odds ratios (ORs) of components of metabolic syndrome for nap duration groups.

Components	Nap Duration (min)				
	0	~30	~60	~90	>90
**All**					
Elevated WC	0.98 (0.82, 1.17)	1.00	1.00 (0.86, 1.16)	1.31 (0.94, 1.83)	**1.55 (1.02, 2.35)**
Elevated TG	0.91 (0.74, 1.13)	1.00	0.93 (0.78, 1.09)	**1.48 (1.04, 2.11)**	1.25 (0.79, 2.00)
Reduced HDL-C	1.14 (0.94, 1.39)	1.00	0.97 (0.82, 1.16)	1.14 (0.78, 1.68)	1.23 (0.77, 1.96)
Elevated BP	1.01 (0.81, 1.26)	1.00	0.97 (0.82, 1.15)	1.12 (0.76, 1.65)	1.12 (0.68, 1.85)
Elevated FPG	1.01 (0.83, 1.24)	1.00	0.94 (0.80, 1.11)	0.93 (0.64, 1.35)	**1.59 (1.02, 2.47)**
**Female**					
Elevated WC	1.05 (0.83, 1.33)	1.00	1.07 (0.86, 1.34)	1.31 (0.80, 2.15)	**2.12 (1.22, 3.70)**
Elevated TG	1.07 (0.78, 1.47)	1.00	1.04 (0.77, 1.40)	0.85 (0.38, 1.91)	1.73 (0.81, 3.67)
Reduced HDL-C	1.21 (0.96, 1.53)	1.00	1.01 (0.81, 1.25)	1.10 (0.66, 1.83)	1.11 (0.61, 2.03)
Elevated BP	1.06 (0.76, 1.48)	1.00	1.09 (0.80, 1.48)	1.04 (0.47, 2.31)	0.63 (0.19, 2.09)
Elevated FPG	1.07 (0.82, 1.39)	1.00	1.03 (0.81, 1.32)	1.08 (0.59, 1.97)	**2.53 (1.41, 4.53)**
**Male**					
Elevated WC	0.91 (0.68, 1.20)	1.00	0.93 (0.76, 1.13)	1.28 (0.81, 2.01)	1.11 (0.62, 2.01)
Elevated TG	0.77 (0.58, 1.03)	1.00	0.87 (0.71, 1.06)	**1.87 (1.21, 2.91)**	1.09 (0.61, 1.94)
Reduced HDL-C	0.93 (0.62, 1.40)	1.00	0.89 (0.67, 1.20)	1.16 (0.64, 2.11)	1.32 (0.63, 2.76)
Elevated BP	0.95 (0.71, 1.27)	1.00	0.93 (0.75, 1.14)	1.14 (0.73, 1.79)	1.48 (0.82, 2.67)
Elevated FPG	0.99 (0.73, 1.35)	1.00	0.87 (0.69, 1.08)	0.84 (0.52, 1.35)	0.97 (0.51, 1.86)

Data were indicated as ORs (95% CIs of ORs) which were calculated by multivariate logistic regression models, adjusting for age, affiliation, marital status, position level, smoking status, drinking status, physical activity, mood symptoms, night sleep duration hours, bedtime at night, use of sleeping medicine, sleep quality, and gender as appropriate. ORs, odds ratios; CIs, confidence intervals; WC, waist circumference; TG, triglycerides; HDL-C, high-density lipoprotein cholesterol; BP, blood pressure; FPG, fasting plasma glucose.

## Supplementary Materials

The following are available online at https://www.mdpi.com/1660-4601/17/12/4268/s1, Figure S1. The association between nap duration and prevalence of metabolic syndrome by three logistic regression models.; Figure S2. The dose-response relationship between nap duration and metabolic syndrome by gender.; Table S1. Characteristics of included participants and excluded participants.; Table S2. Characteristics of participants according to nap duration.; Table S3. Sensitivity analyses of adjustment for more variables, multiple imputation, multilevel model, and propensity score stratifying.

## Abbreviations

SMS	Short Messaging Service;
HDL-C	high-density lipoprotein cholesterol;
PHQ-2	Patient Health Questionnaire-2
GAD-2	General Anxiety Disorder-2
PSQI	Pittsburgh sleep quality index
SD	standard deviation
OR	odds ratio
CIs	confidence intervals
RCS	restricted cubic spline
WC	waist circumference
TG	triglycerides
SBP	systolic blood pressure
DBP	diastolic blood pressure
FPG	fasting plasma glucose
BP	blood pressure
