# Supplementary material for: Association of Nap Duration after Lunch with Prevalence of Metabolic Syndrome in a Chinese Government Employee Population
Source: Int J Environ Res Public Health. 2020 Jun 15;17(12):4268. doi: 10.3390/ijerph17124268 (PMC7344757; doi:10.3390/ijerph17124268)
Supplement: Supplementary file 1 [file ijerph-17-04268-s001.pdf]

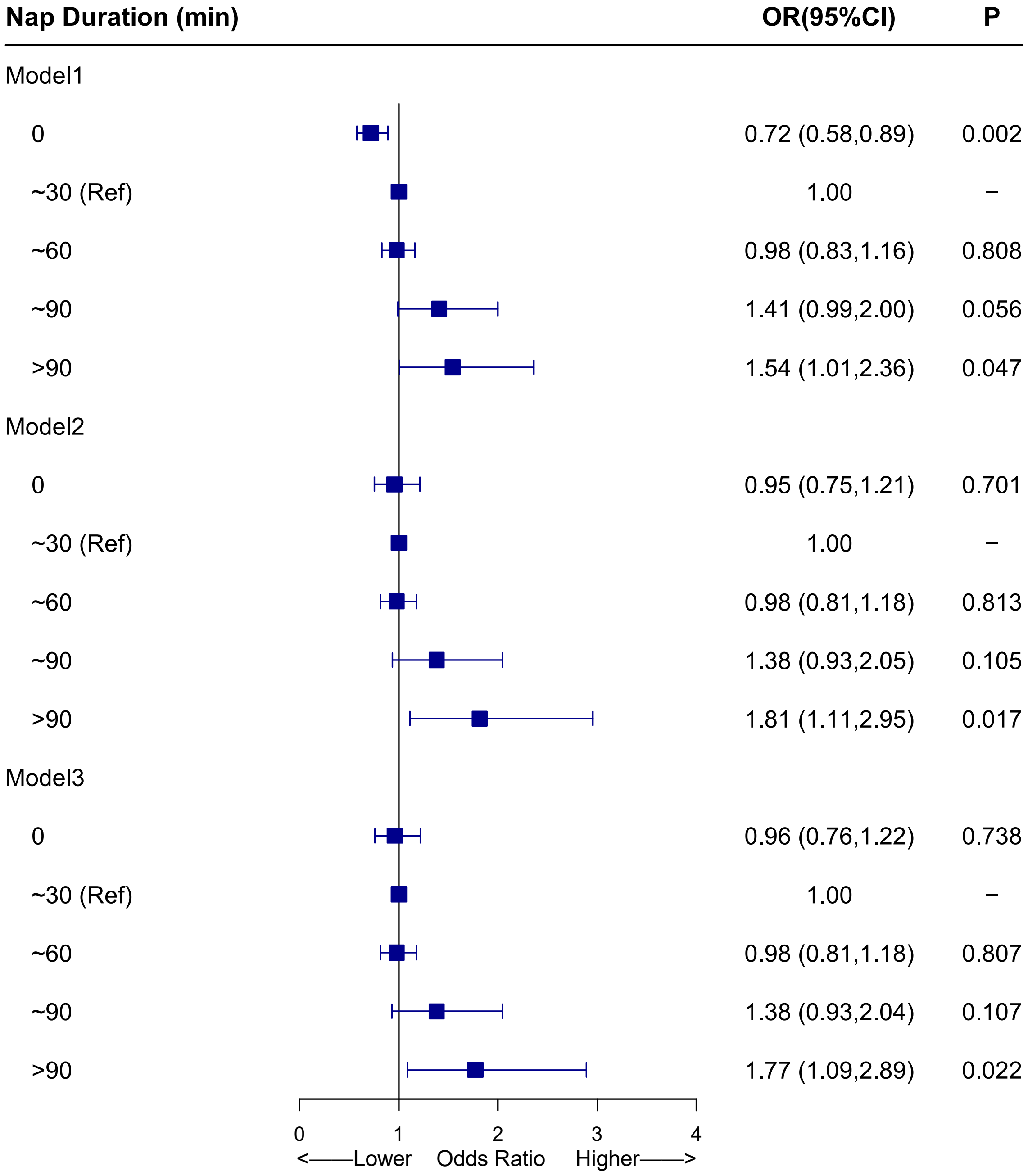

Figure S1. The association between nap duration and prevalence of metabolic syndrome by three logistic regression models.

Model 1 was unadjusted. Model 2 was adjusted for demographic factors, lifestyle habits, and mood symptoms. Model 3 was additionally adjusted for sleep-related characteristics. Small squares represent the point estimates of odds ratios and horizontal lines represent 95% CIs. Min, minutes; OR, odds ratio; CI, confidence interval; Ref, reference.

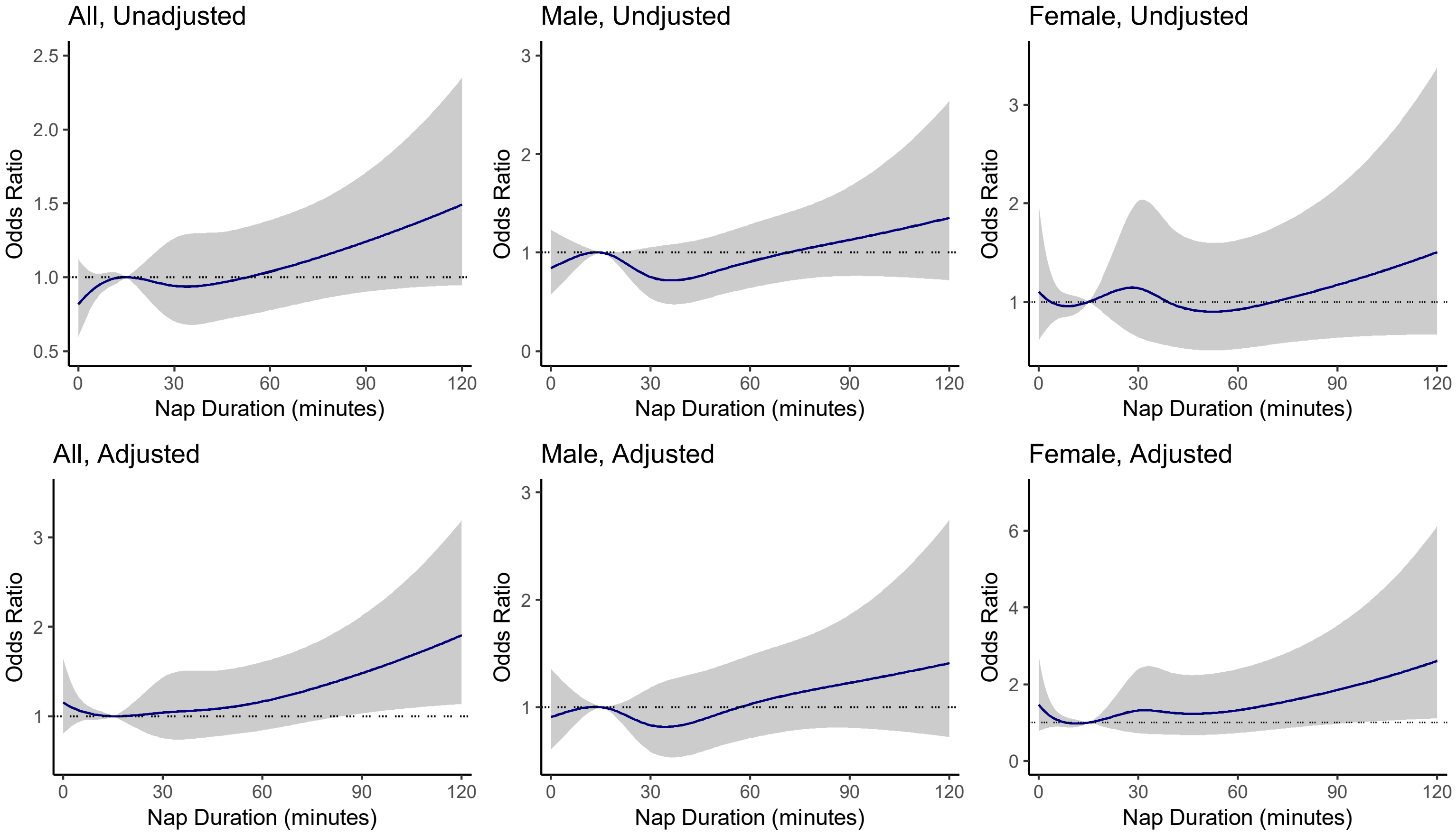

Figure S2. The dose-response relationship between nap duration and metabolic syndrome by gender.

Logistic regression models with 5 knots restricted cubic spline functions were adjusted for age, affiliation, marital status, position level, smoking status, drinking status, physical activity, mood symptoms, night sleep duration hours, bedtime at night, use of sleeping medicine, sleep quality, and gender as appropriate. The references were set to 15 minutes. The shadows represent 95% confidence intervals of odds ratios.

Table S1. Characteristics of included participants and excluded participants.

Characteristics	Included participants (Available data on variables)	Excluded participants (Missing data on variables)	***p***
N	5129	1266	
Age (years), mean (SD)	39.43 (9.31)	39.67 (9.38)	0.405
Gender, female (%)	3025 (59.0)	635 (50.2)	<0.001
Affiliations (%)			<0.001
Government department	370 (7.2)	103 (11.5)	
Public institution	3432 (66.9)	558 (62.3)	
State-owned enterprise	1327 (25.9)	235 (26.2)	
Marital status (%)			0.337
Married/cohabitating	4349 (84.8)	774 (86.6)	
Unmarried	634 (12.4)	100 (11.2)	
Divorced/widowed	146 (2.8)	20 (2.2)	
Position levels (%)			0.022
Primary title/staff member/clerk	1857 (36.2)	343 (39.1)	
Intermediate title/section level	2103 (41.0)	371 (42.3)	
Senior title/division level or above	1169 (22.8)	164 (18.7)	
Current smoking (%)	612 (11.9)	59 (11.5)	0.829
Current drinking (%)	354 (6.9)	348 (39.5)	<0.001
Participating physical activity (%)	2985 (58.2)	536 (59.9)	0.363
Having mood symptoms (%)	526 (10.3)	101 (11.5)	0.295
Night sleep duration(hours), mean (SD)	7.55 (1.05)	7.61 (1.08)	0.145
Using sleeping medication (%)	186(3.6)	44(5.0)	0.062
Night sleep quality (%)			0.189
Good	2221 (43.3)	356 (40.4)	
Fair	2335 (45.5)	430 (48.8)	
Bad	573 (11.2)	95 (10.8)	
Nap duration (minutes), mean (SD)	31.02 (27.43)	31.73 (29.11)	0.486
WC (cm), mean (SD)	78.66 (9.68)	81.77 (10.19)	<0.001
TG (mmol/L), mean (SD)	1.41 (1.39)	1.67 (1.50)	<0.001
HDL-C (mmol/L), mean (SD)	1.42 (0.32)	1.44 (0.38)	0.029
SBP (mmHg), mean (SD)	116.92 (13.74)	118.88 (13.50)	<0.001
DBP (mmHg), mean (SD)	71.18 (10.55)	73.17 (11.01)	<0.001
FPG (mmol/L), mean (SD)	5.37 (1.00)	5.42 (1.04)	0.124

Data were indicated as mean (standard deviation) or number (percentage). *P* was calculated using *t*-test for continuous variables and chi-square test for categorical variables. SD, standard deviation; WC, waist circumference; TG, triglycerides; HDL-C, high-density lipoprotein cholesterol; SBP, systolic blood pressure; DBP, diastolic blood pressure; FPG, fasting plasma glucose.

Table S2. Characteristics of participants according to nap duration.

Characteristics	Nap Duration (minutes)					*p*
	0	~30	~60	~90	>90	
N	953	2312	1551	193	120	
Age (years), mean (SD)	38.06 (9.36)	40.44 (9.27)	38.91 (9.16)	39.05 (9.24)	37.93 (9.76)	<0.001
Gender, female (%)	680 (71.4)	1362 (58.9)	817 (52.7)	98 (50.8)	68 (56.7)	<0.001
Affiliations (%)						<0.001
Government department	57 (6.0)	172 (7.4)	114 (7.4)	17 (8.8)	10 (8.3)	
Public institution	750 (78.7)	1429 (61.8)	1016 (65.5)	147 (76.2)	90 (75.0)	
State-owned enterprise	146 (15.3)	711 (30.8)	421 (27.1)	29 (15.0)	20 (16.7)	
Marital status (%)						<0.001
Married/cohabitating	787 (82.6)	2001 (86.5)	1303 (84.0)	170 (88.1)	88 (73.3)	
Unmarried	144 (15.1)	241 (10.4)	209 (13.5)	18 (9.3)	22 (18.3)	
Divorced/widowed	22 (2.3)	70 (3.0)	39 (2.5)	5 (2.6)	10 (8.3)	
Position levels (%)						<0.001
Primary title/staff member/clerk	412 (43.2)	788 (34.1)	535 (34.5)	74 (38.3)	48 (40.0)	
Intermediate title/section level	368 (38.6)	950 (41.1)	659 (42.5)	77 (39.9)	49 (40.8)	
Senior title/division level or above	173 (18.2)	574 (24.8)	357 (23.0)	42 (21.8)	23 (19.2)	
Current smoking (%)	96 (10.1)	233 (10.1)	219 (14.1)	39 (20.2)	25 (20.8)	<0.001
Current drinking (%)	48 (5.0)	161 (7.0)	118 (7.6)	20 (10.4)	7 (5.8)	0.037
Participating physical activity (%)	439 (46.1)	1429 (61.8)	951 (61.3)	109 (56.5)	57 (47.5)	<0.001
Having mood symptoms (%)	113 (11.9)	229 (9.9)	145 (9.3)	28 (14.5)	11 (9.2)	0.081
Night sleep duration(hours), mean (SD)	7.61 (1.14)	7.54 (1.00)	7.53 (1.01)	7.49 (1.17)	7.58 (1.28)	0.365
Using sleeping medication (%)	38(4.0)	91(3.9)	46(3.0)	5(2.6)	6(5.0)	0.384
Night sleep quality (%)						<0.001

Characteristics	Nap Duration (minutes)					p
	0	~30	~60	~90	>90	
Good	374 (39.2)	1001 (43.3)	706 (45.5)	89 (46.1)	51 (42.5)	
Fair	420 (44.1)	1088 (47.1)	691 (44.6)	80 (41.5)	56 (46.7)	
Bad	159 (16.7)	223 (9.6)	154 (9.9)	24 (12.4)	13 (10.8)	
Nap duration (minutes), mean (SD)	0.00 (0.00)	20.30 (11.98)	52.68 (8.73)	84.86 (7.70)	117.17 (17.50)	<0.001
WC (cm), mean (SD)	76.90 (9.42)	78.73 (9.58)	79.22 (9.78)	80.67 (9.63)	80.72 (10.58)	<0.001
TG (mmol/L), mean (SD)	1.27 (1.14)	1.42 (1.32)	1.44 (1.51)	1.81 (2.14)	1.51 (1.25)	<0.001
HDL-C (mmol/L), mean (SD)	1.46 (0.31)	1.42 (0.32)	1.41 (0.32)	1.36 (0.30)	1.37 (0.31)	<0.001
SBP (mmHg), mean (SD)	114.62 (13.59)	117.22 (13.76)	117.52 (13.46)	118.87 (14.25)	118.74 (15.28)	<0.001
DBP (mmHg), mean (SD)	69.17 (10.06)	71.64 (10.58)	71.48 (10.39)	72.55 (11.34)	72.19 (12.48)	<0.001
FPG (mmol/L), mean (SD)	5.28 (0.72)	5.42 (1.05)	5.37 (1.12)	5.35 (0.65)	5.34 (0.92)	0.013
Metabolic syndrome (%)	128 (13.4)	411 (17.8)	271 (17.5)	45 (23.3)	30 (25.0)	<0.001

Data were indicated as mean (standard deviation) or number (percentage). *P* was calculated using *t*-test for continuous variables and chi-square test for categorical variables. WC, waist circumference; SD, standard deviation; TG, triglycerides; HDL-C, high-density lipoprotein cholesterol; SBP, systolic blood pressure; DBP, diastolic blood pressure; FPG, fasting plasma glucose.

Table S3. Sensitivity analyses of adjustment for more variables, multiple imputation, multilevel model, and propensity score stratifying.

Nap Duration (minutes)	OR	95% CI of OR	*p*
**Further adjusted for family history and diet frequency***			
0	0.95	0.75-1.21	0.697
~30	1.00	-	-
~60	0.98	0.81-1.18	0.805
~90	1.40	0.94-2.08	0.097
>90	**1.79**	**1.08-2.96**	**0.024**
**Multiple imputation method**			
0	1.00	0.81-1.25	0.970
~30	1.00	-	-
~60	1.00	0.84-1.20	0.969
~90	1.39	1.00-1.95	0.054
>90	**1.69**	**1.06-2.69**	**0.028**
**Multilevel model****			
0	0.94	0.74-1.19	0.615
~30	1.00	-	-
~60	0.97	0.81-1.17	0.749
~90	1.34	0.91-1.99	0.141
>90	**1.73**	**1.06-2.82**	**0.028**
**Propensity score stratifying analysis*****			
0~90	1.00	-	-
>90	**1.65**	**1.08-2.52**	**0.020**

OR, odds ratio; CI, confidence interval.

*Multivariable logistic regression model was adjusted for age, gender, affiliation, marital status, position level, smoking status, drinking status, physical activity, mood symptoms, night sleep duration hours, bedtime at night, use of sleeping medicine, sleep quality, and further adjusted for family history and diet frequency factors, including family history of hypertension, cancer, and diabetes mellitus, weekly frequency of eating coarse cereals, meat, poultry, aquatic products, egg products, vegetables, dairy products, fruits, and dessert.

**In the multilevel model the individuals were level 1, and organizations were level 2.

***Propensity scores were calculated by logistic regression with dependent variable dichotomized by whether the nap duration was greater than 90 minutes and were divided into 6 strata.
